# A genomic exploration of the early evolution of extant cats and their sabre-toothed relatives

**DOI:** 10.12688/openreseurope.13104.2

**Published:** 2021-06-21

**Authors:** Michael V Westbury, Ross Barnett, Marcela Sandoval-Velasco, Graham Gower, Filipe Garrett Vieira, Marc de Manuel, Anders J Hansen, Nobuyuki Yamaguchi, Lars Werdelin, Tomas Marques-Bonet, M Thomas P Gilbert, Eline D Lorenzen

**Affiliations:** 1 The GLOBE Institute, University of Copenhagen, Øster Voldgade 5-7, Copenhagen, Denmark; 2 Institute of Evolutionary Biology (UPF-CSIC), PRBB, Dr. Aiguader 88, 08003 Barcelona, Spain; 3 Institute of Tropical Biodiversity and Sustainable Development, Universiti Malaysia Terengganu, 21030 Kuala Nerus, Terengganu, Malaysia; 4 Department of Palaeobiology, Swedish Museum of Natural History, Box 50007, SE-104 05 Stockholm, Sweden; 5 Catalan Institution of Research and Advanced Studies (ICREA), Passeig de Lluís Companys, 23, 08010, Barcelona, Spain; 6 CNAG-CRG, Centre for Genomic Regulation (CRG), Barcelona Institute of Science and Technology (BIST), Baldiri i Reixac 4, 08028 Barcelona, Spain; 7 Institut Català de Paleontologia Miquel Crusafont, Universitat Autònoma de Barcelona, Edifici ICTA-ICP, c/ Columnes s/n, 08193 Cerdanyola del Vallès, Barcelona, Spain; 8 Department of Natural History, NTNU University Museum, Norwegian University of Science and Technology (NTNU), NO-7491 Trondheim, Norway

**Keywords:** Smilodon, ancient DNA, genomics, gene flow, Felidae, palaeogenome, phylogeny

## Abstract

**Background:** The evolutionary relationships of Felidae during their Early–Middle Miocene radiation is contentious. Although the early common ancestors have been subsumed under the grade-group *Pseudaelurus, *this group is thought to be paraphyletic, including the early ancestors of both modern cats and extinct sabretooths.

**Methods:** Here, we sequenced a draft nuclear genome of *Smilodon populator,* dated to 13,182 ± 90 cal BP, making this the oldest palaeogenome from South America to date, a region known to be problematic for ancient DNA preservation. We analysed this genome, together with genomes from other extinct and extant cats to investigate their phylogenetic relationships.

**Results:** We confirm a deep divergence (~20.65 Ma) within sabre-toothed cats. Through the analysis of both simulated and empirical data, we show a lack of gene flow between *Smilodon* and contemporary Felidae.

**Conclusions:** Given that some species traditionally assigned to *Pseudaelurus* originated in the Early Miocene ~20 Ma, this indicates that some species of *Pseudaelurus* may be younger than the lineages they purportedly gave rise to, further supporting the hypothesis that *Pseudaelurus* was paraphyletic.

## Plain language summary

Here we sequenced the genome of the extinct sabre-toothed cat *Smilodon populator.* By comparing this genome to those of living cat species as well as another extinct sabre-toothed cat *Homotherium latidens,* we were able to assess their evolutionary relationships to one another. We show that not only are sabre-toothed and extant cats very divergent from one another, but there were also highly divergent species within sabre-toothed cats. This high level of divergence within sabre-toothed cats makes it difficult to place the common ancestor of all cat species based on just the fossil record. Moreover, we were able to show that it was very unlikely that sabre-toothed cats hybridised with any living cat species.

## Introduction

Based on the fossil record, the origin of Felidae, colloquially referred to as cats, is well resolved to the Middle-Late Oligocene (~30–27 million years ago (Ma)) ^1^. However, the subsequent radiation of Felidae in the Early–Middle Miocene (~23–15 Ma) is less well understood, encompassing the evolution and radiation from the common ancestor of a number of species subsumed under the paraphyletic grade-group *Pseudaelurus*, which was widely distributed across Europe, Asia, and North America ^1^. The putative paraphyly of *Pseudaelurus* is exemplified by the fact that it likely includes not only the early ancestors of modern cats (Felinae) but also those of the extinct sabretooths (Machairodontinae).

In contrast to the early radiation of Felidae, the phylogenetic relationships among extant cats are relatively well understood; extant cats are believed to share a most recent common ancestor in the Late Miocene (~11 Ma) ^2^. However, despite this, there is uncertainty surrounding the constituents of the long-stem lineage of Felinae after its split from Machairodontinae (which extends through the Early and Middle Miocene) until its radiation in the Late Miocene. The evolutionary relationships within Machairodontinae are even less well understood, and increased knowledge of the evolutionary relationships both between Felinae and Machairodontinae, as well as within Machairodontinae itself, would provide important insights to help resolve the complex early radiation of Felidae.

The last surviving members of the Machairodontinae belonged to the genus *Smilodon* (tribe Smilodontini). While once widespread across the continents of North America ( *S. fatalis*) and South America ( *S. populator*), the genus went extinct ~10 thousand years ago (kya) ^3, 4^. *Smilodon* are not known further north than southernmost Canada (~42 °N), and their range extended until the tip of the South American continent (~53 °S). Apart from a few anomalous mass occurrences in tar pits (e.g. Rancho La Brea, USA and Talara, Peru), *Smilodon* is relatively rare in the fossil record, which is not uncommon for an apex carnivore. Moreover, when it is present, there are only limited remains ^5^.

The evolutionary relationships of *Smilodon* to other extinct and extant large cats are not resolved. An early ancient DNA study using both mitochondrial and nuclear DNA placed *Smilodon* within Felinae ^6^. However, this was later shown to likely reflect contaminant DNA from a domestic cat ^7^. A later study using complete mitochondrial genomes found *Smilodon* to be highly divergent from both Felinae (with an estimated divergence of ~20 Ma), and another extinct Machairodontinae lineage ( *Homotherium* (tribe Homotheriini)), diverging ~18 Ma ^8^. However, conclusions based exclusively on a single maternally inherited locus can be biased by interspecific hybridisation and incomplete lineage sorting, which has been well-documented in living cats ^9^.

To date, only three studies present authentic DNA from *Smilodon* ^8, 10, 11^, all of which is mitochondrial DNA. Although there is a large collection from Rancho La Brea, retrieving endogenous DNA from the material is not considered feasible ^7^, greatly restricting the number of specimens available for DNA study. The relative absence of studies of *Smilodon* likely in part reflects their rarity in the fossil record outside of tar pits. In addition, as *Smilodon* are assumed to have been mixed wood-edge ambush predators ^12^, the majority of fossils are found in open-air sites with poor DNA preservation; cave sites (which afford better preservation) are rare. Finally, the detrimental effects of temperature at equatorial and near-equatorial latitudes further exacerbate DNA preservation.

To elucidate the evolutionary relationship of *Smilodon* to extant and extinct Felidae, and to gain insights into the early radiation of Felidae, we sequenced a draft nuclear genome of a single *Smilodon populator* individual from the Ultima Esperanza region of Chile. The specimen was radiocarbon dated to 13,182 ± 90 calibrated years before present (cal BP), and represents the oldest palaeogenome from South America, a region in which ancient DNA preservation is expected to be limited ^13^.

## Results and discussion

Using an African lion assembly as the reference genome, we successfully mapped a draft nuclear genome of a single *Smilodon populator* individual to an average genome-wide coverage of ~0.7x, with an average read depth of ~2x. We achieved this using a combination of both target enrichment and shotgun sequencing. We performed target enrichment using baits created from either DNA extracted from a modern lion (whole-genome capture), or based on the exome annotations of the lion reference genome (exome capture). The discrepancy between genome-wide coverage and average read-depth likely reflects the use of captured data, and lack of a closely related reference genome. If we were to have a conspecific reference genome, we would expect a more even genome-wide coverage, more comparable to the read depth.

We sequenced approximately 800 million reads from four independently constructed libraries ( Table 1). Investigations into mapped reads showed high levels of fragmentation (average read length <90bp for all libraries) and high rates of C-T transitions across the reads (Figure S1, *Extended data* ^14^), both typical of authentic ancient DNA. Comparisons among libraries showed that the capture experiments did yield significant levels of endogenous DNA, but were not highly different from the shotgun data but had lower levels of complexity. Moreover, although the exome capture was able to increase the relative coverage of the exome, it also included a lot of non-coding whole genomic data ( Table 1). 

**Table 1.** *Smilodon* mapping results. WGC - whole genome capture, SE - single end, PE - paired end.

Library name	BU19_5	BU23_1	Smi745_1	Smi745_2
**Library type**	Exome capture	WGC	WGC/Shotgun mix	Shotgun
**# SE reads**	145,712,209	7,856,010	125,060,258	64,850,185
**# PE reads**	164,381,221	309,399,989	-	-
**# PE reads merged**	146,380,424	278,659,340	-	-
**# Retained reads**	313,124,333	342,949,720	121,988,548	60,427,680
**# Mapped reads**	6,070,092	6,474,904	5,191,598	7,136,656
**Proportion duplicates**	0.893	0.830	0.731	0.100
**Endogenous proportion**	0.019	0.019	0.043	0.118
**Exome coverage**	1.237	0.217	0.605	0.272
**Genome coverage**	0.171	0.234	0.139	0.179
**Average read length (bp)**	68.7	88.3	65.4	61.3

To investigate the topological placement of *Smilodon*, we computed a neighbour-joining (NJ) tree using genome-wide transversion pairwise distances, rooted using *Crocuta crocuta* (spotted hyena). Similar to the mitochondrial genome, we found *Smilodon* and *Homotherium* to be sister taxa, and Machairodontinae to be sister taxon to all extant cats (Figure S2, *Extended data* ^14^). Support for this topology was high; we repeated this analysis independently for the 100 longest scaffolds (27.2Mb - 5.3Mb), and found identical placement of *Smilodon* for each scaffold.

We further tested the closer affinity of *Smilodon* and *Homotherium* to each other, relative to any living Felinae, using D-statistics ^15^ to test for topology. For this, we took advantage of the topological input requirement for the D-statistics test ([[[H1, H2], H3], Outgroup]). We computed the D-statistic with *Smilodon* and *Homotherium* as sister taxa (i.e. in the H1 and H2 positions) (Table S1, *Extended data* ^14^) *,* each species of Felinae as H3, and the spotted hyena as Outgroup, and compared this to the D-statistic and significance from 0 (Z-score) when placing *Smilodon* and *Homotherium* paraphyletically (i.e. in the H1 and H3 positions) (Table S2, *Extended data* ^14^). We clearly see a much higher D-statistic (D= 0.54-0.67, Z= 286.6-442.5) when placing the sabre-toothed cats parapyletically, compared to when they are placed as sister taxa (D= 0.12-0.20, Z= 39.2-67.7). A high D-statistic could be interpreted as gene flow between either H1 and H3, or between H2 and H3. However, another possible explanation could be the incorrect input topology. Although paraphyly of the sabre-toothed cats followed by gene flow could explain the observed pattern, a more likely explanation for the higher D-statistic when placing the sabre-tooths paraphyletically, would be the monophyly of the sabre-tooths as seen in our NJ tree. This result further supports *Smilodon* and *Homotherium* as more closely related to one another, than either are to any extant cat species.

**Figure 1.**
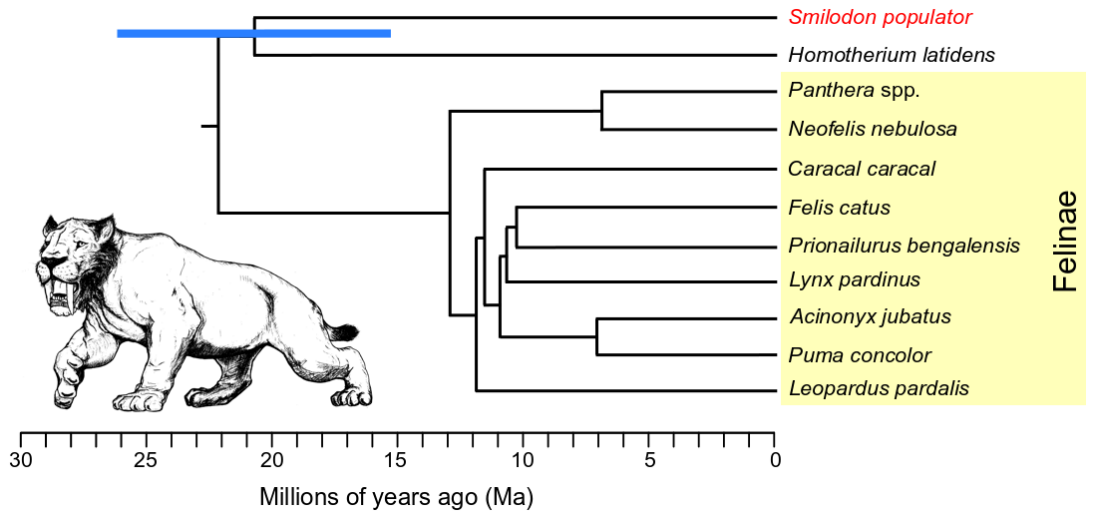
Dated Felidae phylogenetic tree based on genome-wide pairwise F2 statistics, calibrated using an average genetic drift rate estimated from within Felinae (yellow shading). Blue bar shows 95% confidence interval for the divergence between the *Smilodon* (red) *Homotherium* lineages, calculated from the F2 standard error. *Smilodon* illustration by Binia De Cahsan and included with permission.

 To further contextualise this relationship, we built a dated phylogenetic tree ( Figure 1). However, due to the low coverage of our *Smilodon* genome, traditional methods for phylogeny dating are likely unsuitable. Therefore, we devised a method to overcome this by computing pairwise genetic drift distances using F-statistics (in our case F2) (Table S3, *Extended data* ^14^) ^16^. The pairwise F2 statistics were built into an unrooted NJ tree (Figure S3, *Extended data* ^14^). The relationships recovered in this tree were the same as those obtained using the transversional genetic distances.

To calibrate our phylogenetic tree, we estimated the average rate of genetic drift between all pairs of genera within Felinae based on the F2 results and previously calculated divergence dates ^17^. We made a number of assumptions about our data, including (i) a strict molecular clock, (ii) constant population sizes through time, and (iii) that Felinae drift rates are similar to those in Machairodontinae.

Using the F2 statistics and average drift rate, we estimated a divergence date between Machairodontinae and Felinae of ~22.1 Ma ( Figure 1), similar to the estimate of ~22.5 Ma calculated using a high-coverage exome dataset ^17^. Although the correlation between these two results was not unexpected, as our tree was calibrated using the average within Felinae drift calculated using the divergence times of Barnett *et al.* ^17^, their similar divergence estimates suggest extrapolating Felinae drift rates to other subfamilies of Felidae is valid.

We further tested for the robustness of this method by both recalculating the divergence times within Felinae, and by downsampling two Felinae species ( *Caracal caracal -* caracal and *Prionailurus bengalensis* - leopard cat) to ~0.7x. We recovered similar divergence estimates to those previously reported, and to those produced without downsampling, providing confidence in the use of this methodology for low-coverage genomes (Tables S3 and S4, *Extended data* ^14^). Using this methodology, we estimated the divergence date of *Smilodon*/ *Homotherium* to be ~20.65 Ma (95% CI 26.07-15.25 Ma) ( Figure 1; Table S5, *Extended data* ^14^). Furthermore, although based on low-coverage data and a number of data assumptions, the congruence of our results with previous mitogenome-based estimates from the same individuals ^8^, despite the use of different data and calibration methods, further adds confidence to our method of phylogenetic assessment.

Species traditionally assigned to *Pseudaelurus* originated in the Early Miocene, ~20 Ma. Given the deep divergence between Machairodontinae and Felinae, as well as within Machairodontinae, this indicates that some species of *Pseudaelurus* are younger than the lineages they purportedly gave rise to. This is strong support for the hypothesis that *Pseudaelurus* as a grade-group is paraphyletic. Furthermore, current phylogenies using well-known characters (such as length of upper canines and presence of serrations or crenulations) resolve the Smilodontini-Homotheriini divergence to only ~11-10 Ma, while older machairodonts constitute a stem lineage of uncertain relationship ^18^. Therefore, given the problematic nature of machairodont phylogenetics, the new molecular information analysed here provides an important reference point for identifying morphologically Smilodontini or Homotheriini characters in specimens from >11 Ma, to help resolve machairodont phylogeny back to the early Miocene.

We next investigated the evolutionary relationships between *Smilodon* and Felinae by searching for signatures of gene flow, using two independent methods (f3 ^16^ and D3 ^19^). The results were assessed using simulated data (Tables S6, S7, and S8, *Extended data* ^14^). As lineages leading to *Smilodon* and *Homotherium* diverged relatively soon after the Machairodontinae/Felinae split, we were able to test for ancient gene flow (up to 20 Ma) between *Smilodon* or *Homotheirum* and stem Felinae. Ancient gene flow may have prevented the diverging lineages in early Felidae from accumulating obvious morphological differences, thus preventing the confident phylogenetic placement of lineages during this time period.

Moreover, we tested for more recent gene flow events between either *Smilodon* or *Homotherium* and the entire *Panthera* big cat lineage (all *Panthera* species grouped together), due to their potential size overlap, as well as with single species that may have more recently met *Smilodon* due to spatial proximity (i.e. from South America: *Panthera onca* (jaguar), *Puma concolor* (puma), and *Leopardus pardalis* (ocelot)).

We did not find any indication of gene flow between any of the lineages tested, regardless of method (Tables S6 and S7, *Extended data* ^14^). The lack of more recent gene flow events is not surprising, due to the relatively ancient divergence of Machairodontinae and Felinae. However, the lack of ancient gene flow signatures do not necessarily mean that gene flow was not present during the early divergence of these lineages. Rather, it may result from the inadequacy of the methods in uncovering such events.

We assessed the power of the D3 statistic to detect gene flow, which occurred at different times (20 Ma-50 kya), using simulated data. When using a simple demographic model of constant population size, mutation rate, and recombination rate on error-free simulated data, we detect significant levels of gene flow back to ~16 Ma (Table S8, *Extended data* ^14^). However, empirical data do not always behave in such a simple manner, and a variety of factors may influence the results of the D3 statistic. These include, but are not restricted to: violations of the infinite sites model, different mutation rates across lineages, ancestral populations structure, and introgression with unsampled lineages ^19^. However, our results suggest that D3 may be suitable for highly divergent lineages with ancient gene flow events and therefore violations of the infinite sites model may not be as problematic.

Thus, although we are unable to exclude the possibility of ancient gene flow during the early radiation of Felidae, we are somewhat more confident that there was no more recent gene flow between either *Smilodon* or *Homotherium* and Felinae within the last 16 Ma years. If very ancient gene flow had occurred prior to 16 Ma, it is likely that recombination and genetic drift would have either highly fragmented the introgressed regions, or completely removed them from contemporary Felinae individuals. This would render the regions too small to be detected with current methods, which use genome-wide summary statistics or regions of phylogenetic incongruence with known evolutionary relationships, to infer gene flow.

Our study exemplifies how even a draft palaeogenome from an extinct species can provide important information into their evolutionary history. Through the sequencing of a single *Smilodon populator* genome, we provide insights into Felidae’s early radiation in the Early–Middle Miocene (~23 - 15 Ma), which could not be uncovered using genetic data from extant species alone.

## Methods

### Sample information

Specimen ZMA20.042 is from the Naturalis museum, Leiden, the Netherlands, and was radiocarbon dated to 11,335±30 uncalibrated years before present (2-sigma range of 13,269-13,095 calibrated years before present) (Stafford: UCIAMS-142836). We calibrated the radiocarbon date using Calib v7.04 ^20^ using the int13.14c calibration curve. It has been identified as a left tibia of *Smilodon populator* and is part of the Kruimel collection, an assortment of megafaunal remains recovered from the Última Esperanza region of Chile, most likely from the site of Cueva del Milodón. This and other specimens of *Smilodon* from the Kruimel Collection were described and figured by McDonald and Werdelin (2018); specimen ZMA20.042 is presented in Figure 4.6D ^5^. We additionally included genomic information from another extinct sabre-toothed cat, 12 extant cat species, and the spotted hyena ( Table 2).

**Table 2.** List of the additional species included in this study. Original sources and accession codes for all raw reads used in this study can be found in Table S9 (see *Extended data* ^14^).

Species name:	Common name:
*Felis catus*	Domestic cat
*Prionailurus bengalensis*	Leopard cat
*Lynx pardinus*	Iberian lynx
*Acinonyx jubatus*	Cheetah
*Caracal caracal*	Caracal
*Neofilis nebulosa*	Clouded leopard
*Panthera uncia*	Snow leopard
*Panthera onca*	Jaguar
*Panthera pardus*	Leopard
*Panthera leo*	Lion
*Crocuta crocuta*	Spotted hyena
*Homotherium latidens*	Scimitar-toothed cat
*Puma concolor*	Puma
*Leopardus pardalis*	Ocelot

### Ancient DNA extraction, library preparation, and sequencing

Samples of cortical bone were taken from the long bone element (approx. 1 cm ^3^) using a Dremel drill, and reduced to powder in a Mikrodismembrator. Two independent DNA extractions were performed as described in Orlando *et al*. ^21^ in a dedicated ancient DNA laboratory, with negative controls. We built each DNA extract and negative control into genomic libraries using the NEB E6070 kit following a modified version of the protocol used by Vilstrup *et al.* ^22^. Briefly, the extract (30 µl) was end-repaired and cleaned using a MinElute column, the collected flow-through was adapter-ligated and cleaned using a QiaQuick column, and the adapter fill-in reaction was performed on the flowthrough. To complete the library build, we performed a final incubation at 37°C (30 min) followed by inactivation overnight at -20°C.

For each library, we performed two independent indexing PCR amplifications (Veriti thermal cycler, Applied Biosystems) in a 50 µl volume reaction, using 25 µl of library, 25 µl PCR master mix, and 12 cycles of PCR reactions. The final concentrations in the PCR master mix were 1.25 U AccuPrime™ Pfx DNA Polymerase (Invitrogen, Cat # 12344-024), 1x AccuPrime™ Pfx reaction mix (Invitrogen, Cat # 12344-024), 0.4 mg/ml BSA, 120 nM primer InPE, 120 nM of a multiplexing indexing primer containing a unique six-nucleotide index code (Illumina – sequences TGCAGG, CGATGA, GCGAGA, or CAGCAC). PCR cycling conditions consisted of an initial denaturation step at 95°C for 2 min, followed by 12 cycles of 95°C denaturation for 15 s, 60°C annealing for 30 s, and 68°C extension for 30 s, and a final extension step at 68°C for 7 min. Indexed libraries were checked for presence of DNA on a 2% agarose gel before purification using the QIAquick column system (Qiagen, Cat # 28104) and quantification on an Agilent 2100 BioAnalyzer. Quantified libraries were communally pooled in equimolar ratios and sequenced as 100 bp single-end reads on an Illumina HiSeq2000 platform at the Danish National High-Throughput Sequencing Centre and 100 bp paired-end reads on an Illumina Hiseq2000 at BGI Copenhagen.

### Genome capture

We assessed the shotgun-sequenced libraries for endogenous content and selected the libraries with the highest levels of endogenous DNA for two sets of capture experiments. The first set used biotinylated RNA probes transcribed from fresh DNA extracted from a modern lion, for the purpose of enriching the entire nuclear genome (whole-genome capture). The second method used biotinylated RNA baits, assembled based on the exonic annotations of lion genomic data ^23^ (exome capture). Both types of baits were generated by Arbor Biosciences (Ann Arbor, MI, USA) and carried out using the myBaits target enrichment kit and the instructions described in manual V3.

After capture and cleanup, enriched libraries were re-amplified for further sequencing using either a Phusion polymerase (New England Biolabs, Cat # M0530S) or a KAPA HiFi HotStart polymerase (Roche, Cat # KK2801 07959052001) with primers IS5_reamp.P5 and IS6_reamp.P7 over 14 cycles ^24^. We quantified the resultant enriched libraries on a TapeStation 2200 instrument and sequenced them on an Illumina Hiseq2000 at the Danish National High-throughput Sequencing Centre.

### Data processing pipeline

Post-sequencing read processing of the *Smilodon populator* was performed using the PALEOMIX v1.2.5 pipeline ^25^. Adaptor sequence removal and trimming of low-quality bases (BaseQ < 5 or Ns) was done with AdapterRemoval v2.0.0 ^26^. This step also removed all reads shorter than 30 bp in length or with more than 50 bp of missing data. Trimmed reads were mapped against an African lion reference genome (NCBI Accession JAAVKH000000000 ^27^) with BWA-MEM v0.7.5a ^28^, utilising default parameters. PCR duplicates were identified and filtered based on the 5'-end mapping coordinate using Picard v2.18.0. *GATK* v3.8.0 ^29^ was used to perform an *indel* realignment step to adjust for increased error rates at the end of short reads in the presence of *indels*. In the absence of a curated dataset of *indels*, this step relied on a set of *indels* identified in the specific sample being processed. Read damage patterns were assessed and base quality scores recalibrated around read terminal damage patterns using mapDamage v2.0.5 ^30, 31^.

Data processing of the extant Felidae species, excluding *Puma concolor* and *Leopardus pardalis*, followed the same pipeline with the following minor adjustments; no minimum read length cut-off or missing data cut-off was applied during the adapter trimming step, and bases were not recalibrated using mapDamage. These steps were removed as the data from the extant species would not display the highly fragmented and damage patterns found in ancient DNA. The *Puma concolor* and *Leopardus pardalis* samples had Illumina adapter and short sequences trimmed using skewer 0.2.2 ^32^ but followed the same protocol as the other extant species for the rest of the processing steps.

### Neighbour-joining tree

To build a NJ phylogenetic tree, we computed an identity by state distance matrix considering only transversion differences using ANGSD v0.921 ^33^, and specifying the following parameters; call the consensus base (-doIBS 2), minimum mapping and quality of 30 (-minmapq 30, -minq 30), only include a site if all individuals are covered (-minInd), remove secondary alignments (-remove_bads 1), only include reads that map to a single location (-uniqueonly 1), compute major and minor alleles based on genotype likelihoods (-domajorminor 1), remove transitions (-rmtrans 1), print all sites (-minminor 0), and use the GATK algorithm to compute genotype likelihoods (-GL 2) and only include scaffolds over 1Mb in length (-rf). After filtering, 221,350,529 sites remained.

We further checked for support of the genome-wide topology by building a distance matrix for the 100 longest scaffolds independently, resulting in 100 independent distance matrices based on 171,281 - 3,344,878 sites. The resultant distance matrices were converted into NJ trees using fastME v2.1.5 ^34^.

### D-statistics topology test

To investigate the closer relationship of *Smilodon* and *Homotherium* to each other relative to other extant cat species, we implemented a D-statistics test for topology ^35^. Although D-statistics is most commonly used to find evidence of gene flow, it can also be used to test for phylogenetic relationships. This test takes advantage of the fact that D-statistics relies on a predefined four-taxon topology as input [[[H1,H2],H3],outgroup]. A high D-score is most commonly used to infer post-divergence gene flow, but it can also be caused by more recent common ancestry brought about by an incorrect predefined topology.

Taking the latter into account, we performed a number of D-statistics tests including the topologies: [[[ *Smilodon*, *Homotherium*], extant cat], *Crocuta crocuta*], [[[ *Smilodon*, extant cat], *Homotherium*], *Crocuta crocuta*], and [[[ *Homotherium*, extant cat], *Smilodon*], *Crocuta crocuta*]. In this test, ‘extant cat’ was replaced with each living Felidae species included in this study. We used ANGSD v0.921 ^33^ to perform the D-statistics with the following parameters; -minmapq 30 -minq 30, -minind 15, -remove_bads 1, -uniqueonly 1, -domajorminor 1, -rmtrans 1, -GL 2, calculate D in a block size of 1Mb (-blocksize), and the spotted hyena as the ancestral/outgroup sequence (-anc).

### Dated phylogeny

We computed the consensus haploid base calls (-dohaplocall 2) for all scaffolds greater than 1MB in length using ANGSD, specifying the following parameters; minimum mapping and quality of 30 (-minmapq 30, -minq 30), only include a site if all individuals are covered (minInd), remove secondary alignments (-remove_bads 1), only include reads that map to a single location (-uniqueonly 1), compute major and minor alleles based on genotype likelihoods (-domajorminor 1), remove transitions (-rmtrans 1), print all sites (-minminor 0), and use the GATK algorithm to compute genotype likelihoods (-GL 2). After filtering, 268,152,250 sites remained.

We converted the haploid call file into a PLINK file format using the haplo2plink command from the ANGSD toolsuite. Using the resultant PLINK file, we calculated F2 statistics ^16^ for each pairwise combination of our *Smilodon populator* individual, *Homotherium latidens*, and 12 Felinae species using the popstats.py script ^36^. We used genetic drift calculated via F-statistics as opposed to absolute pairwise distance as it should be more suitable for ancient DNA data ^37^. From these pairwise F2 comparisons, we built a distance matrix that was converted into a newick tree file using PHYLIP v3.696 neighbor ^38^ for visualisation.

From the distance matrix, we computed the Felinae average rate of genetic drift (F2) to be 0.000305 per million years. For this, we calculated the average F2 between pairwise comparisons of all genera within Felinae that had divergence time estimates available in Barnett *et al.* ^17^. These included *Acinonyx*, *Caracal*, *Felis*, *Lynx*, *Neofelis*, *Panthera*, and *Prionailurus*, and resulted in 21 comparisons (Table S3, *Extended data* ^14^). We used the formula of (F2 × 0.5)/divergence time to estimate the rate of genetic drift per million years for all 21 comparisons. We calculated the mean of these 21 comparisons, giving us the Felinae mean rate of genetic drift per million years. This mean rate was used in conjunction with the previously calculated average F2 to calibrate the tree including the *Smilodon populator*, *Homotherium latidens,* puma, and ocelot.

To test for the robustness of our method to low-coverage data, we downsampled both *Caracal* and *Prionailurus* to comparable coverage to *Smilodon populator* (~0.7x) using SAMtools v1.6 ^39^, and recomputed the F2 statistics. We used the same average rate of genetic drift to estimate the divergence of these genera from their closest relatives (i.e. *Prionailurus* - *Felis*, and *Caracal* - *Acinonyx*/ *Felis*/ *Lynx*). For this analysis we assumed a known species tree and divergences within Felinae previously found in Barnett *et al.* ^17^, a genome-wide constant mutation/drift rate, and no variation in drift rates between lineages. Uncertainty around the divergence between *Smilodon* and *Homotherium* was calculated using 1.96 x the standard deviation on either side of the F2 statistic. The standard deviation was calculated using the standard error x **√** N (where N = number of blocks using for jackknifing).

### Assessing gene flow

We implemented two independent analyses to test for the presence of signs of gene flow between Machairodontinae and Felinae. We computed F3 statistics ^16^ to assess whether there were any signals of gene flow between a predefined triplet [[A,B],C] using the same PLINK file computed above for the F2 statistics. We used popstats.py ^36^ for five independent triplet combinations, which we present as ((A,B),C) in the subsequent text. We placed *Smilodon* and *Homotherium* in the A and B positions, while alternating C.

First, we investigated signs of very ancient gene flow between Machairodontinae and Felinae by specifying all extant Felinae as one population. Next, we looked for gene flow with the *Panthera* spp. big cats by specifying all *Panthera* as a single population. Finally, we computed F3 three times independently to test for signs of very recent gene flow between Machairodontinae and either of three extant cat species occupying South America, which may have come into contact with *Smilodon* based on geography ( *Panthera onca* (jaguar), *Puma concolor* (puma), and *Leopardus pardalis* (ocelot)).

To complement this analysis, we also computed D3 statistics ^19^ on the same five triplet comparisons. For this, we also produced a haplocall file in ANGSD using the same filtering parameters as above, but using a random base call (-dohaplocall 1), as opposed to the consensus base call, while specifying the same additional parameters specified above. The resultant haploid output was then converted into a geno file and run through the popgenWindows.py python script. We ran the popgenWindows.py script using default parameters, specifying a window size of 1MB, and the minimum number of sites per window as 1kb. From this output, we calculated D3 for each window independently by applying the equation [[BC-AC]/[BC+AC]]. Mean values, standard deviations, and significance from 0 were measured in R v3.6.0 using the pnorm function ^40^.

### Evaluating D3 for detecting ancient gene flow

To evaluate the adequacy of the D3 method for detecting very ancient gene flow events (up to 20 Ma), we simulated three diploid sequences representing *Homotherium* (A), *Smilodon* (B), and a Felinae species (C), using msprime ^41^. As input, we estimated the average transversion mutation rate within Felidae using the average pairwise distance between *Homotherium* and Felinae and divided this by two before multiplying it by the Machairodontinae/Felinae divergence time calculated above (22.1 Ma). We computed pairwise comparisons between *Homotherium* and each Felinae species included in the study using ANGSD and took the average pairwise distance from these comparisons. We excluded the *Smilodon* from this calculation due to the lower quality of the genome.

To calculate the pairwise distances we used the consensus identity by state parameter (-doIBS 2) in ANGSD and applied the following filters; -minmapq 30, -minq 30, -minind 15, -uniqueonly 1, -remove_bads 1, -domajorminor 1, -rmtrans 1, -minminor 0, -makematrix 1, and only included scaffolds >1MB in length. This gave us an average pairwise distance of 0.01207, which we converted into an average transversion mutation rate of 2.730769e-10 per year. We converted this to a generational mutation rate using a generation time calculated for lions of 6.5 years ^42^; if the generation time of the investigated species differed from that of lion, the mutation rate would adjust accordingly, and we therefore expect minimal impact of this parameter on the final results, especially as we ran the simulations specifying years as opposed to number of generations. For the recombination rate, we used a previously published sex-averaged recombination rate of 1.9 cM/Mb (1.9e-8) ^43^.

Using the above information, we ran five independent simulations, each consisting of 2,000 1 Mb windows with a constant effective population size of 40,000 individuals, a generation time of 6.5 years, the above-mentioned mutation and recombination rates, a 5% pulse of migration (m=0.05), and a different timing of the migration pulse for each of the five runs (20 Ma, 18 Ma, 17 Ma, 16 Ma, 15 Ma, 10 Ma, 5 Ma, 50 kya). The scripts for these simulation runs can be found on GitHub. We calculated the D3 statistic from this output using the tskit toolkit ^44^ using the d3.py script. Significance was calculated as it was done for the empirical data above.

## Data availability

### Underlying data

NCBI BioProject: Raw sequencing reads for the *Smilodon* individual. Accession number PRJNA691254; https://www.ncbi.nlm.nih.gov/bioproject/691254.

### Extended data

Zenodo: A genomic exploration of the early evolution of extant cats and their sabre-toothed relatives - extended data. https://doi.org/10.5281/zenodo.4922450 ^14^.

This project contains the following extended data:

- Westbury et al extended data.pdf (Supplementary Figures S1-S3 and Tables S1-S9)

Data are available under the terms of the Creative Commons Attribution 4.0 International license (CC-BY 4.0).
