# Supplementary material for: A genomic exploration of the early evolution of extant cats and their sabre-toothed relatives [version 2; peer review: 2 approved]
Source: Open Res Eur. Author manuscript; Available in PMC 2022 Jan 28. (PMC7612286)
Supplement: extended data [file EMS129480-supplement-extended_data.pdf]

Supplementary figures

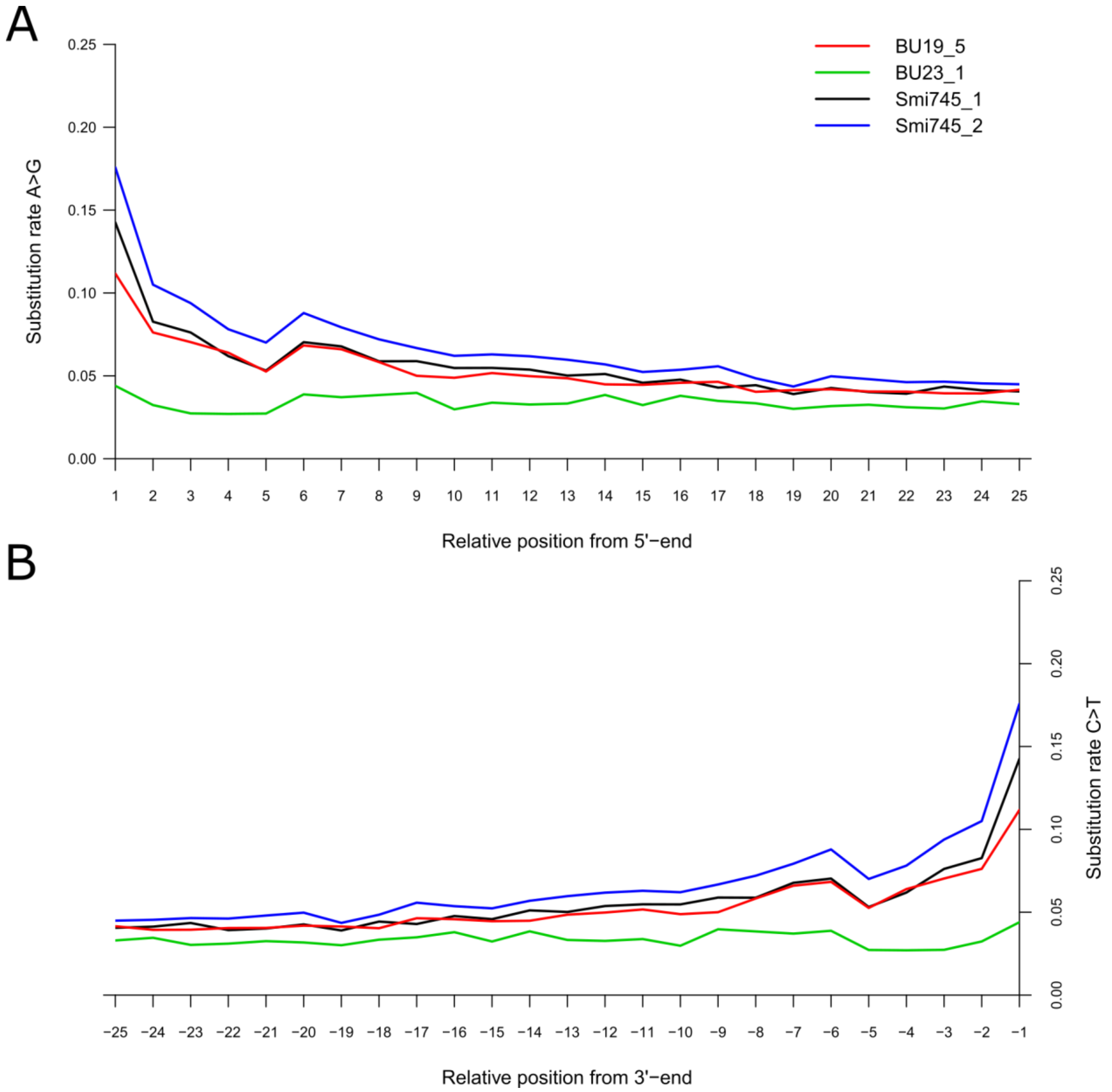

Supplementary figure S1: Levels of ancient DNA damage in the four libraries used to construct the *Smilodon populator* draft genome. (A) Rate of A to G substitutions relative to the 5’ end of the read. (B) Rate of C to T substitutions relative to the 3’ end of the read.

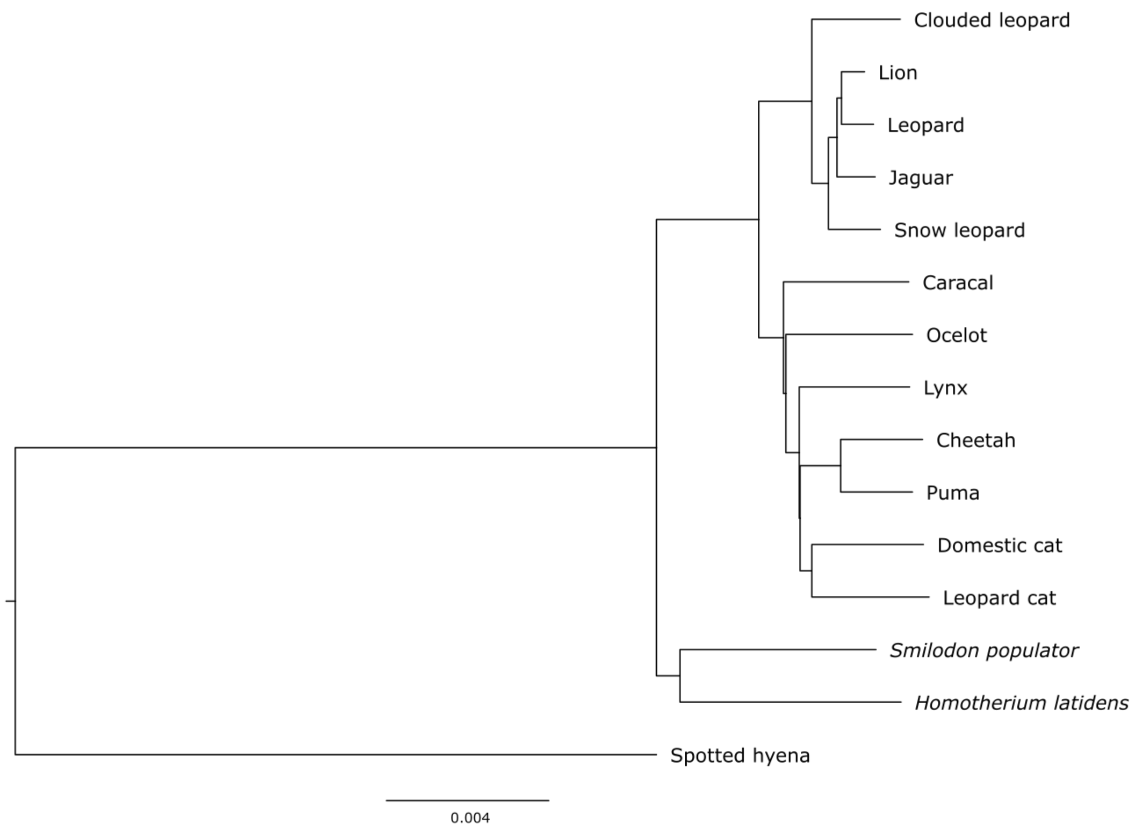

Supplementary figure S2: Neighbour-joining (NJ) tree constructed using genome-wide transversion pairwise distances, rooted using the spotted hyena (*Crocuta crocuta*). Scale bar represents the proportion of differences between sequences.

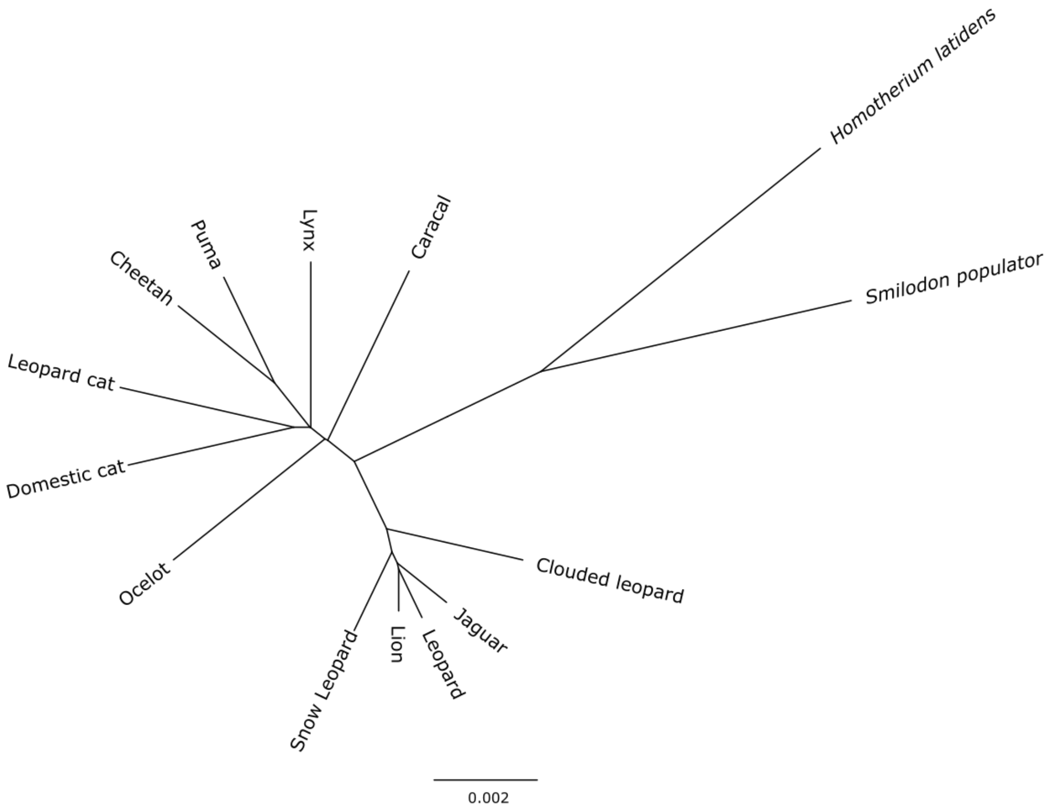

Supplementary figure S3: Neighbour-joining (NJ) tree constructed using genetic drift distances calculated using pairwise F2 -statistics. Scale bar represents the proportion of differences between sequences.

Supplementary tables

**H1**	**H2**	**H3**	**D-statistic**	**Standard Error**	**Z-score**
Smilodon	*Homotherium*	Lion	-0.2024	0.0030	-67.69
Smilodon	*Homotherium*	Leopard	-0.1874	0.0030	-62.39
Smilodon	*Homotherium*	Jaguar	-0.1853	0.0030	-61.54
Smilodon	*Homotherium*	Snow leopard	-0.1780	0.0030	-59.34
Smilodon	*Homotherium*	Clouded leopard	-0.1629	0.0030	-54.05
Smilodon	*Homotherium*	Lynx	-0.1230	0.0031	-40.13
Smilodon	*Homotherium*	Ocelot	-0.1217	0.0031	-39.42
Smilodon	*Homotherium*	Cheetah	-0.1216	0.0031	-39.34
Smilodon	*Homotherium*	Puma	-0.1212	0.0031	-39.45
Smilodon	*Homotherium*	Caracal	-0.1210	0.0031	-39.20
Smilodon	*Homotherium*	Cat	-0.1208	0.0030	-39.95
Smilodon	*Homotherium*	Leopard cat	-0.1207	0.0030	-39.69

Supplementary table S1: D-statistics results when placing *Smilodon* and *Homotherium* monophyletically.

H1	H2	H3	D-statistic	Standard Error	Z-score
Homotherium	Lion	*Smilodon*	-0.5398	0.0019	-286.56
Homotherium	Leopard	*Smilodon*	-0.5425	0.0018	-296.74
Homotherium	Jaguar	*Smilodon*	-0.5428	0.0019	-290.98
Homotherium	Snow leopard	*Smilodon*	-0.5444	0.0018	-297.45
Homotherium	Clouded leopard	*Smilodon*	-0.5490	0.0018	-298.41
Homotherium	Lynx	*Smilodon*	-0.5606	0.0019	-300.92
Homotherium	Ocelot	*Smilodon*	-0.5610	0.0019	-301.52
Homotherium	Cheetah	*Smilodon*	-0.5613	0.0019	-302.34
Homotherium	Puma	*Smilodon*	-0.5612	0.0019	-302.26
Homotherium	Caracal	*Smilodon*	-0.5604	0.0018	-302.99
Homotherium	Cat	*Smilodon*	-0.5617	0.0018	-306.86
Homotherium	Leopard cat	*Smilodon*	-0.5618	0.0018	-303.95
Smilodon	Lion	*Homotherium*	-0.6691	0.0015	-442.49
Smilodon	Leopard	*Homotherium*	-0.6626	0.0015	-429.25
Smilodon	Jaguar	*Homotherium*	-0.6615	0.0015	-429.38
Smilodon	Snow leopard	*Homotherium*	-0.6585	0.0016	-420.54
Smilodon	Clouded leopard	*Homotherium*	-0.6535	0.0016	-417.07
Smilodon	Lynx	*Homotherium*	-0.6395	0.0016	-397.97
Smilodon	Ocelot	*Homotherium*	-0.6390	0.0016	-395.76
Smilodon	Cheetah	*Homotherium*	-0.6392	0.0016	-395.61
Smilodon	Puma	*Homotherium*	-0.6389	0.0016	-390.09
Smilodon	Caracal	*Homotherium*	-0.6381	0.0017	-386.04
Smilodon	Cat	*Homotherium*	-0.6391	0.0016	-402.73
Smilodon	Leopard cat	*Homotherium*	-0.6392	0.0016	-398.00

Supplementary table S2: D-statistics results when placing *Smilodon* and *Homotherium* parapyletically.

Genus 1	Genus 2	Divergence (Ma)	Recalculated divergence (Ma)	F2	Rate/million years
Neofelis	*Panthera*	8.32	6.82	0.004161	0.000250
Neofelis	*Felis*	14.08	13.94	0.008505	0.000302
Neofelis	*Prionailurus*	14.08	13.84	0.008443	0.000300
Neofelis	*Caracal*	14.08	12.92	0.007884	0.000280
Neofelis	*Acinonyx*	14.08	13.65	0.008328	0.000296
Neofelis	*Lynx*	14.08	13.22	0.008065	0.000286
Panthera	*Felis*	14.08	12.68	0.007732	0.000275
Panthera	*Prionailurus*	14.08	12.58	0.007675	0.000273
Panthera	*Caracal*	14.08	11.66	0.007112	0.000253
Panthera	*Acinonyx*	14.08	12.38	0.007554	0.000268
Panthera	*Lynx*	14.08	11.96	0.007296	0.000259
Felis	*Prionailurus*	8.16	10.21	0.006230	0.000382
Felis	*Caracal*	11.34	11.76	0.007174	0.000316
Felis	*Acinonyx*	9.66	11.10	0.006774	0.000351
Felis	*Lynx*	9.19	10.64	0.006493	0.000353
Prionailurus	*Caracal*	11.34	11.72	0.007148	0.000315
Prionailurus	*Acinonyx*	9.66	11.06	0.006746	0.000349
Prionailurus	*Lynx*	9.19	10.60	0.006465	0.000352
Caracal	*Acinonyx*	11.34	11.52	0.007029	0.000310
Caracal	*Lynx*	11.34	11.11	0.006777	0.000299
Acinonyx	*Lynx*	9.66	10.53	0.006425	0.000333
**Average**					0.000305

Supplementary table S3: Genus level pairwise F2 comparisons, divergence dates, and genome-wide rates of genetic drift per million years. “Average” indicates the mean value of all individual genome-wide rates of genetic drift. Divergence dates are those based on results from Barnett et al 2020 [1].

			Subsample		High coverage		
**Genus 1**	**Genus 2**	**Actual divergence**	**Estimated divergence**	**F2**	**Estimated divergence**	**F2**	**Divergence difference**
Caracal	*Acinonyx*	11.34	11.36	0.00693	11.52	0.00703	0.16
	Lynx	11.34	10.88	0.00663	11.11	0.00678	0.23
	Felis	11.34	11.40	0.00695	11.76	0.00717	0.36
Prionailurus	*Felis*	8.16	10.05	0.00613	10.21	0.00623	0.16

Supplementary table S4: Comparison of divergence date estimates to their next closest relatives using the full high coverage data and subsampled 0.7x data from the *Caracal* and *Prionailurus* using an average drift rate of 0.000305. Divergence dates are shown in millions of years. “Actual divergence” is the mean divergence from Barnett et al 2020 [1].

**Genus 1**	**Genus 2**		**Divergence (Ma)**	**F2**
Smilodon	*Homotherium*	Mean	20.65	0.01260
Smilodon	*Homotherium*	Upper 95%	26.06	0.01590
Smilodon	*Homotherium*	Lower 95%	15.25	0.00930
Homotherium	*Felinae*		22.75	0.01388
Smilodon	*Felinae*		21.44	0.01308
Machairodontinae	*Felinae*		22.10	0.01348

Supplementary table S5: Divergence times within Machairodontinae and between Machairodontinae and Felinae based on F2 values and an average genome-wide genetic drift rate of 0.000305. Upper and lower 95% intervals are taken from 1.96x the standard deviation (0.001485) of the F2 calculated between *Smilodon* and *Homotherium.*

A	B	C	F3	SE	Z-score
Smilodon	*Homotherium*	Felinae	0.00434	0.00009	48.29
Smilodon	*Homotherium*	*Panthera*	0.00595	0.00012	49.77
Smilodon	*Homotherium*	Jaguar	0.00682	0.00014	47.18
Smilodon	*Homotherium*	Ocelot	0.00782	0.00017	46.36
Smilodon	*Homotherium*	Puma	0.00788	0.00017	47.36

Supplementary table S6: F3 statistics results based on the topology [[A,B],C]. SE shows the standard error and the Z-score shows the number of SE the F3 is away from 0. A negative F3 indicates gene flow while positive shows inconclusive results.

A	B	C	D3	SD	p-value
Smilodon	*Homotherium*	Felinae	-0.00004	0.00677	0.49
Smilodon	*Homotherium*	*Panthera*	0.00544	0.00740	0.07
Smilodon	*Homotherium*	Jaguar	0.00491	0.00769	0.10
Smilodon	*Homotherium*	Ocelot	-0.00413	0.00894	0.18
Smilodon	*Homotherium*	Puma	-0.00358	0.00759	0.17

Supplementary table S7: D3 statistics results based on the topology [[A,B],C]. D3 shows the average D3 value taken from a non-overlapping sliding window size of 1Mb. SD is the standard deviation and significance from 0 is estimated by calculating a p-value assuming a normal distribution. A p-value less than 0.05 is considered significant. A significantly negative D3 value shows gene flow between B and C while a significantly positive D3 shows admixture between A and C.

A	B	C	Date of gene flow	D3	SD	p-value
Homotherium	*Smilodon*	Felinae species	20Ma	-0.0002	0.0044	0.467046
Homotherium	*Smilodon*	Felinae species	18Ma	-0.0026	0.0045	0.127785
Homotherium	*Smilodon*	Felinae species	17Ma	-0.0035	0.0047	0.063969
Homotherium	*Smilodon*	Felinae species	16Ma	-0.0048	0.0049	0.023450
Homotherium	*Smilodon*	Felinae species	15Ma	-0.0057	0.0050	0.010962
Homotherium	*Smilodon*	Felinae species	10Ma	-0.0114	0.0065	0.000248
Homotherium	*Smilodon*	Felinae species	5Ma	-0.0171	0.0085	0.000029
Homotherium	*Smilodon*	Felinae species	50kya	-0.0227	0.0105	0.000007

Supplementary table S8: D3 statistics results based on simulations using the topology [[A,B],C] with predefined gene flow between B and C at different time periods after divergence. The labels given to the taxon A,B, and C are based on the results of the current study but could represent any arbitrary species with the same divergence times, mutation rates, and recombination rates. D3 shows the average D3 value taken from a non-overlapping sliding window size of 1Mb. SD is the standard deviation and significance from 0 is estimated by calculating a p-value assuming a normal distribution. A p-value less than 0.05 is considered significant. A significantly negative D3 value shows gene flow between B and C while a significantly positive D3 shows admixture between A and C.

**Species name:**	**Source:**	**Accession code:**
Felis catus	99 Lives Cat Genome Sequencing Initiative	NCBI SRA accession code: SRR2224864
Prionailurus bengalensis	[1]	NCBI Bioproject accession code: PRJNA649572
Lynx pardinus	[2]	European nucleotide archive accession code: ERA562804
Acinonyx jubatus	[3]	NCBI SRA accession code: SRS1123638
Caracal caracal	[1]	NCBI SRA sample accession code: SAMN15096300
Neofilis nebulosa	[4]	NCBI SRA sample accession code: SAMN14352199
Panthera uncia	[5]	NCBI SRA accession code: SRR836372
Panthera onca	[6]	NCBI SRA sample accession code: SAMN05907657
Panthera pardus	[7]	NCBI SRA accession code: SRR3041424
Panthera leo	[5]	NCBI SRA accession code: SRR836361
Crocuta crocuta	[8]	NCBI Bioproject accession code: PRJNA554753
Homotherium latidens	[1]	NCBI Bioproject accession code: PRJNA649760
Puma concolor	[9]	NCBI SRA accession code:SRR6467080
Leopardus pardalis	[10]	NCBI SRA accession code: SRR6071643

Supplementary table S9: Genbank accession codes and the corresponding sources of the extant cat short read genomic data used in this study.

Supplementary references:

1. Barnett, R., Westbury, M.V., Sandoval-Velasco, M., Vieira, F.G., Jeon, S., Zazula, G., Martin, M.D., Ho, S.Y.W., Mather, N., Gopalakrishnan, S., *et al.* (2020). Genomic Adaptations and Evolutionary History of the Extinct Scimitar-Toothed Cat, *Homotherium latidens*. Curr. Biol. *30*, 1–8.

2. Abascal, F., Corvelo, A., Cruz, F., Villanueva-Cañas, J.L., Vlasova, A., Marcet-Houben, M., Martínez-Cruz, B., Cheng, J.Y., Prieto, P., Quesada, V., *et al.* (2016). Extreme genomic erosion after recurrent demographic bottlenecks in the highly endangered Iberian lynx. Genome Biol. *17,* 251.

3. Dobrynin, P., Liu, S., Tamazian, G., Xiong, Z., Yurchenko, A.A., Krasheninnikova, K., Kliver, S., Schmidt-Küntzel, A., Koepfli, K.-P., Johnson, W., *et al.* (2015). Genomic legacy of the African cheetah, Acinonyx jubatus. Genome Biol. *16,* 277.

4. de Manuel, M., Barnett, R., Sandoval-Velasco, M., Yamaguchi, N., Garrett Vieira, F., Zepeda Mendoza, M.L., Liu, S., Martin, M.D., Sinding, M.-H.S., Mak, S.S.T., *et al.* (2020). The evolutionary history of extinct and living lions. Proc. Natl. Acad. Sci. U. S. A. *117*, 10927–10934.

5. Cho, Y.S., Hu, L., Hou, H., Lee, H., Xu, J., Kwon, S., Oh, S., Kim, H.-M., Jho, S., Kim, S., *et al.* (2013). The tiger genome and comparative analysis with lion and snow leopard genomes. Nat. Commun. *4*, 2433.

6. Figueiró, H.V., Li, G., Trindade, F.J., Assis, J., Pais, F., Fernandes, G., Santos, S.H.D., Hughes, G.M., Komissarov, A., Antunes, A., *et al.* (2017). Genome-wide signatures of complex introgression and adaptive evolution in the big cats. Sci Adv *3,* e1700299.

7. Kim, S., Cho, Y.S., Kim, H.-M., Chung, O., Kim, H., Jho, S., Seomun, H., Kim, J., Bang, W.Y., Kim, C., *et al.* (2016). Comparison of carnivore, omnivore, and herbivore mammalian genomes with a new leopard assembly. Genome Biol. *17,* 211.

8. Westbury, M.V., Hartmann, S., Barlow, A., Preick, M., Ridush, B., Nagel, D., Rathgeber, T., Ziegler, R., Baryshnikov, G., Sheng, G., *et al.* (2020). Hyena paleogenomes reveal a complex evolutionary history of cross-continental gene flow between spotted and cave hyena. Science Advances *6*, eaay0456.

9. Saremi, N.F., Supple, M.A., Byrne, A., Cahill, J.A., Coutinho, L.L., Dalén, L., Figueiró, H.V., Johnson, W.E., Milne, H.J., O'Brien, S.J., *et al.* (2019). Puma genomes from North and South America provide insights into the genomic consequences of inbreeding. Nat. Commun. *10*, 4769.

10. Li, G., Figueiró, H.V., Eizirik, E., and Murphy, W.J. (2019). Recombination-Aware Phylogenomics Reveals the Structured Genomic Landscape of Hybridizing Cat Species. Mol. Biol. Evol. *36*, 2111–2126.
