# Supplementary material for: A genomic exploration of the early evolution of extant cats and their sabre-toothed relatives [version 2; peer review: 2 approved]
Source: Open Res Eur. Author manuscript; Available in PMC 2022 Jan 28. (PMC7612286)
Supplement: Supplementary Material [file EMS129480-supplement-Supplementary_Material.pdf]

Open Peer Review

Current Peer Review Status: ✔ ✔

Version 1

Reviewer Report 19 April 2021

https://doi.org/10.21956/openreseurope.14174.r26653

© **2021 Salesa M.** This is an open access peer review report distributed under the terms of the Creative Commons Attribution License, which permits unrestricted use, distribution, and reproduction in any medium, provided the original work is properly cited.

✔ **Manuel Salesa**
Departamento de Paleobiología, Museo Nacional de Ciencias Naturales-CSIC, Madrid, Spain

In the present work, the authors show the results of the sequencing of a draft nuclear genome of the largest sabre-toothed felid, *Smilodon populator*, with an age of around 13,000 years ago, making this the oldest palaeogenome from South America to date, a region known to be problematic for ancient DNA preservation. The analysis of this genome, together with genomes from other extinct and extant cats, allows the authors to suggest a series of very interesting phylogenetic proposals. Among them, they confirm a deep divergence (around 20.65 Ma) within Machairodontinae, something that was evident from the fossil record, but never stated from palaeogenomics.

I haven't found major concerns about this work. The methodology seems impeccable, the figure is informative, the taxonomic citations are correct, and the discussion is well supported by the data. The style of the text is clear, and the manuscript is correctly written. I just detected a grammatical mistake when citing *S. populator* on page 3, which is cited as "*popular*". Finally, I find the subject treated in the manuscript suitable for its indexing on *Open Research Europe.*

MINOR COMMENTS

Although fossils of Early and Middle Miocene felids are known since the XIX century, our understanding of their phylogenetic relationships has not changed greatly over this time. This was in part due to the fact that those fossils of primitive felids, mostly fragmentary dental and postcranial material, show a very similar morphology to that of extant species, and only sabre-toothed taxa exhibit evident differences in their elongated and flattened upper canines or their huge carnassial teeth. In the present paper, Westbury *et al*. have sequenced the genome of one of the most iconic sabre-toothed felids, the giant *Smilodon populator*, and then they compared it to those of extant species of Felidae, as well as to that of another extinct sabre-toothed species, the relatively slender and cursorial *Homotherium latidens*. The results were expectable but very interesting: they not only show the strong divergence between Machairodontinae (sabre-toothed felids) and Felinae (extant felids), but also between the two analysed species of sabre-toothed felids, *S. populator* and *H. latidens*. These two derived forms were the last sabre-toothed felids that

were present in the faunas, and both were extinct as recently as around 28,000–11,000 years. *Smilodon* and *Homotherium* illustrate two very different ways to be a sabre-toothed felid: *S. populator*, included in a lineage known as Smilodontini, was a strong-built animal, with short and powerful limbs and very elongated upper canines, whereas *H. latidens* was a more slender predator, with relatively long limbs (the hind limbs much shorter than the forelimbs) and shorter canines. Thus, the divergence found by Westbury *et al*. fits well with the idea that the sabre-toothed felids developed at least two different morphotypes very early in their evolution, a robust, forest-dweller type illustrated by the Middle Miocene *Pseudaelurus quadridentatus* and the Late Miocene *Promegantereon ogygia*, and a slenderer model, probably occupying more open environments, whose first well-known representative is the Late Miocene *Machairodus aphanistus*. These two lineages evolved in parallel, refining each of the morphotypes, occupying different habitats thanks to different locomotor adaptations, thus allowing the presence in the faunas of two large predators through habitat segregation.

Concerning the early divergence between sabre-toothed felids and felines, the present work by Westbury *et al*. points towards a date of around 22.1–22.5 Ma. This is interesting, as we have little data on the felids of that moment, but all the available fossils suggest that the split between felines and macairodontines occurred at that time. There are several European localities with fossils slightly younger than that age (around 18 Ma) that can be identified as early felines, as their dentitions exhibit short canines of a rounded section, although they also show some very primitive features such as the presence of m2; and besides these forms, there are other fossils that show an incipient development of the macairodontine model illustrated by the laterally flattened upper canines of *Pseudaelurus quadridentatus*. Now seems clear that this latter genus should be considered as the first known sabre-toothed felid, as it has laterally flattened upper canines, a relatively robust first metacarpal, and an overall postcranial anatomy very similar to that of the younger *Promegantereon ogygia* (Beaumont, 1975[1]; Salesa *et al*., 2010[2]). Nevertheless, as the present work by Westbury *et al*. remarks, the genus *Pseudaelurus* has traditionally included felids with evident differences in their dental morphology, both from North America and Eurasia; the paraphyly of this genus was already suggested by Werdelin *et al.* (2010)[3] but this new work reinforces this idea.

And finally, it is very interesting that, although Westbury *et al.* remark that they were unable to exclude the possibility of ancient gene flow during the early radiation of Felidae, they are somewhat more confident that there was no more recent gene flow between either *Smilodon* or *Homotherium* and Felinae within the last 16 Ma. Felinae and Machairodontinae developed a body plan devoted to preying upon different kinds of prey, and their dental and skeletal morphology suggests an early independent evolution of each model. This absence of gene flow also suggests the existence of several ethological and physiological differences between felines and sabre-toothed felids whose trace unfortunately vanished when the latter disappeared.

References

1. de Beaumont G: Recherches sur les Félidés (Mammifères, Carnivores) du Pliocène inférieur des sables à Dinotherium des environs d’Eppelsheim (Rheinhessen). *Archives des Sciences Physiques et Naturelles, Genève*. **28**: 369-405
2. SALESA M, ANTÓN M, TURNER A, ALCALÁ L, et al.: Systematic revision of the Late Miocene sabre-toothed felid Paramachaerodus in Spain. *Palaeontology*. 2010; **53** (6): 1369-1391 Publisher Full Text
3. Werdelin L, Yamaguchi N, Johnson WE, O’Brien SJ: Phylogeny and evolution of cats (Felidae). *Biology and Conservation of Wild Felids - Oxford University Press*. 2010. 59-82

Is the work clearly and accurately presented and does it cite the current literature?
Yes

Is the study design appropriate and does the work have academic merit?
Yes

Are sufficient details of methods and analysis provided to allow replication by others?
Yes

If applicable, is the statistical analysis and its interpretation appropriate?
Yes

Are all the source data underlying the results available to ensure full reproducibility?
Yes

Are the conclusions drawn adequately supported by the results?
Yes

Competing Interests: No competing interests were disclosed.

Reviewer Expertise: Palaeobiology, Systematics, Functional anatomy

I confirm that I have read this submission and believe that I have an appropriate level of expertise to confirm that it is of an acceptable scientific standard.

Author Response 10 Jun 2021
Michael Westbury, The GLOBE Institute, University of Copenhagen, Øster Voldgade 5-7, Copenhagen, Denmark

We would like to thank Manuel Salesa for praising our work and giving a very interesting summary of the results within the context of prior literature. We believe this additional information published alongside our manuscript to be helpful to the reader to better understand the background of the study. We have also corrected the mistake when citing S. populator on page 3, which was cited as “popular”.

Competing Interests: No competing interests were disclosed.

Reviewer Report 08 April 2021

https://doi.org/10.21956/openreseurope.14174.r26645

© 2021 Förster D. This is an open access peer review report distributed under the terms of the Creative Commons Attribution License, which permits unrestricted use, distribution, and reproduction in any medium, provided the original work is properly cited.

Daniel Förster
Evolutionary Genetics Department, Leibniz Institute for Zoo and Wildlife Research (IZW), Berlin, Germany

Westbury *et al.* use nuclear data of a *Smilodon populator* individual to resolve the phylogenetic relationship of this species with another extinct sabre-toothed cat (*Homotherium latidens*), and of the Machairodontinae (here, Smilodon and Homotherium) with the extant cats (Felinae). They illuminate the deeper divergences within this Carnivoran family, i.e. the Felidae.

The study confirms that *Smilodon* and *Homotherium* are sister lineages, previously suggested by mtDNA data, and, as the authors point out, these results have implications for resolving the phylogeny of the machairodonts based on morphological characters. Gene flow between lineages during the Early Miocene radiation of the Felidae was not detected, within machairodonts or between machairodonts and stem Felinae, potentially owing to available data and analytical approaches that were possible. An absence of gene flow between machairodonts and extant cats in the more recent past is not too surprising.

An interesting study illuminating the history of a charismatic group. Unfortunately, perhaps owing to genome coverage, the authors did not examine genomic adaptations as Barnett *et al.* 2020 did for *Homotherium latidens*.

Tables & Figures
It seems unnecessary to avoid any Tables in the main text. For example, having the table of species analysed (Table S10) in the Methods section would benefit clarity, as these data suddenly find mention in the data processing section, as well as being featured in multiple subsequent sections.

The mapping results (Table S1) are summarized in the Results & Discussion section, but the table could feature in the main text as well.

Plain language summary
“assess” rather than “access”?

Introduction
The first sentence reads rather clunky.

Results & Discussion
The start of this section would benefit from more details about (1) baits used for capture (based on data available for the lion; exome capture missing here?), and (2) mapping approach used (i.e. to which species: lion).

Can their approach to detect gene flow be applied to some Felinae (e.g. Panthera) in their dataset to confirm that it does indeed function for more recent events?

Methods
Exome capture used DNA baits? (typo?)

Why was the lion genome used as reference? Why not a genome with higher contiguity? Did this

not potentially result in unnecessary loss of data during filtering (e.g. due to scaffold lengths)?

Is the work clearly and accurately presented and does it cite the current literature?
Yes

Is the study design appropriate and does the work have academic merit?
Yes

Are sufficient details of methods and analysis provided to allow replication by others?
Yes

If applicable, is the statistical analysis and its interpretation appropriate?
Yes

Are all the source data underlying the results available to ensure full reproducibility?
Yes

Are the conclusions drawn adequately supported by the results?
Yes

Competing Interests: No competing interests were disclosed.

Reviewer Expertise: Conservation genetics (incl felids), evolutionary genetics (incl felids), archival and ancient DNA (incl felids).

I confirm that I have read this submission and believe that I have an appropriate level of expertise to confirm that it is of an acceptable scientific standard.

Author Response 10 Jun 2021

Michael Westbury, The GLOBE Institute, University of Copenhagen, Øster Voldgade 5-7, Copenhagen, Denmark

We would like to thank Daniel Förster for taking the time to review our manuscript and for providing useful feedback that we have now incorporated into our manuscript. We have included our responses to each comment below.
Tables & Figures It seems unnecessary to avoid any Tables in the main text. For example, having the table of species analysed (Table S10) in the Methods section would benefit clarity, as these data suddenly find mention in the data processing section, as well as being featured in multiple subsequent sections.
Answer: We have included the table listing the names of the additional species included in this study to the methods section.
The mapping results (Table S1) are summarized in the Results & Discussion section, but the table could feature in the main text as well.
Answer: We have now shifted the mapping results table to the main text.
Plain language summary “assess” rather than “access”?
Answer: Fixed

Introduction The first sentence reads rather clunky.
Answer: We have now removed “a family within Carnivora” to make it smoother to read.
Results & Discussion The start of this section would benefit from more details about (1) baits used for capture (based on data available for the lion; exome capture missing here?), and (2) mapping approach used (i.e. to which species: lion).
Answer: We have now added more information about the baits and mapping approach to this section.
Can their approach to detect gene flow be applied to some Felinae (e.g. Panthera) in their dataset to confirm that it does indeed function for more recent events?
Answer: We have now tested the D3 method on some Felinae species. We find that our method does not detect gene flow between Panthera species. However, we do find significant signs of different levels of gene flow between Panthera (more specifically the snow leopard) and its sister genus, Neofelis (clouded leopard). Although the lack of detection between Panthera is unexpected based on the literature (e.g. Figueiro et al 2017 DOI: 10.1126/sciadv.1700299), this may be due to our strict filtering, artificially making the closely related species look more similar to each other. We note that the results between more distantly related Panthera species are only just not significant (p=0.066). We have included a table with the results here.

Methods Exome capture used DNA baits? (typo?)
Answer: Thank you for finding this. Yes it is a typo and has now been corrected to RNA baits.

Why was the lion genome used as reference? Why not a genome with higher contiguity? Did this not potentially result in unnecessary loss of data during filtering (e.g. due to scaffold lengths)?
Answer: We used the lion as reference genome as the baits were designed with lion sequences and to make results more comparable with our previous study (Barnett et al 2020) where we investigated the Homotherium genome within the context of extant Felinae species which were also all mapped to the same reference genome. Although there are currently other Felinae chromosome level assemblies available, they were not available at the time of the bait design. Despite this, even after filtering of shorter scaffolds, we were still left with 613 scaffolds >1Mb in length equating to 2,107,406,870bp which was more than sufficient for our purposes especially as we either relied on the complete dataset (trees) or 1Mb sliding windows (gene flow analyses).

Competing Interests: No competing interests were disclosed.
